# *BHD* mutations, clinical and molecular genetic investigations of Birt–Hogg–Dubé syndrome: a new series of 50 families and a review of published reports

**DOI:** 10.1136/jmg.2007.054304

**Published:** 2008-01-30

**Authors:** J R Toro, M-H Wei, G M Glenn, M Weinreich, O Toure, C Vocke, M Turner, P Choyke, M J Merino, P A Pinto, S M Steinberg, L S Schmidt, W M Linehan

**Affiliations:** 1 Genetic Epidemiology Branch, Division of Cancer Epidemiology and Genetics, National Cancer Institute, National Institutes of Health, Rockville, Maryland, USA; 2 Basic Research Program, SAIC–Frederick Inc, Frederick, Maryland, USA; 3 Urologic Oncology Branch, Center for Cancer Research, NCI, NIH, Bethesda, Maryland, USA; 4 Dermatology Branch, Center for Cancer Research, NCI, NIH, Bethesda, Maryland, USA; 5 Molecular Imaging Program, Center for Cancer Research, NCI, NIH, Bethesda, Maryland, USA; 6 Laboratory of Pathology, Center for Cancer Research, NCI, NIH, Bethesda, Maryland, USA; 7 Biostatistics and Data Management Section, Center for Cancer Research, NCI, NIH, Bethesda, Maryland, USA

## Abstract

**Background:**

Birt–Hogg–Dubé syndrome (BHDS) (MIM 135150) is an autosomal dominant predisposition to the development of follicular hamartomas (fibrofolliculomas), lung cysts, spontaneous pneumothorax, and kidney neoplasms. Germline mutations in *BHD* are associated with the susceptibility for BHDS. We previously described 51 BHDS families with *BHD* germline mutations.

**Objective:**

To characterise the *BHD* mutation spectrum, novel mutations and new clinical features of one previously reported and 50 new families with BHDS.

**Methods:**

Direct bidirectional DNA sequencing was used to screen for mutations in the *BHD* gene, and insertion and deletion mutations were confirmed by subcloning. We analysed evolutionary conservation of folliculin by comparing human against the orthologous sequences.

**Results:**

The *BHD* mutation detection rate was 88% (51/58). Of the 23 different germline mutations identified, 13 were novel consisting of: four splice site, three deletions, two insertions, two nonsense, one deletion/insertion, and one missense mutation. We report the first germline missense mutation in *BHD* c.1978A>G (K508R) in a patient who presented with bilateral multifocal renal oncocytomas. This mutation occurs in a highly conserved amino acid in folliculin. 10% (5/51) of the families had individuals without histologically confirmed fibrofolliculomas. Of 44 families ascertained on the basis of skin lesions, 18 (41%) had kidney tumours. Patients with a germline *BHD* mutation and family history of kidney cancer had a statistically significantly increased probability of developing renal tumours compared to patients without a positive family history (p = 0.0032). Similarly, patients with a *BHD* germline mutation and family history of spontaneous pneumothorax had a significantly increased greater probability of having spontaneous pneumothorax than BHDS patients without a family history of spontaneous pneumothorax (p = 0.011). A comprehensive review of published reports of cases with *BHD* germline mutation is discussed.

**Conclusion:**

BHDS is characterised by a spectrum of mutations, and clinical heterogeneity both among and within families.

Birt–Hogg–Dubé syndrome (BHDS) (MIM 135150) is an autosomal dominantly inherited genodermatosis that predisposes to the development of cutaneous hamartomas (fibrofolliculomas), kidney neoplasms, lung cysts and spontaneous pneumothorax.^1^ ^2^ We mapped the *BHD* locus to the short arm of chromosome 17(17p11.2).^3^ Subsequently, we found that germline mutations in *BHD* (GenBank accession number AF517523) (also known as FLCN), were associated with the susceptibility for BHDS.^4^ *BHD* is composed of 14 exons. We previously reported 51 BHDS families with germline mutations in *BHD*, and to date more than 40 unique mutations in *BHD* have been reported.^4^^–^16 Germline insertion or deletion of a cytosine in the hypermutable polycytosine (C8) tract in exon 11 of the *BHD* gene has been detected in 53% of BHDS families and is suggested as a mutation “hot spot”.^6^ Most *BHD* germline mutations are frameshift or nonsense mutations that are predicted to truncate the BHD protein, folliculin.^4^^–^16

Two naturally occurring renal cancer syndromes in animals have been described: hereditary multifocal renal cystadenocarcinoma and nodular dermatofibrosis in German shepherd dogs^17^ ^18^ and hereditary renal carcinoma in the Nihon rat.^19^ ^20^ Germline mutations in the *BHD* ortholog genes have been identified in affected animals with these renal cancer syndromes: a missense mutation in exon 7 in the canine *bhd* gene,^21^ and an insertion of a cytosine in a C_5_ tract in the rat *bhd* gene.^22^ Furthermore, a high frequency of loss of heterozygosity (LOH) of the wild type *bhd* allele has been demonstrated in renal tumours in Nihon rats suggesting that *BHD* functions as a tumour suppressor gene.^19^ ^20^

*BHD* is predicted to encode a 579 amino acid protein, folliculin, which is highly conserved among species. BHD mRNA is expressed in a variety of tissues including stromal cells, the distal nephron of the kidney, type I pneumocytes of the lung, and skin and its appendages.^23^ However, reduced expression of *BHD* was seen in renal tumours from patients with BHDS,^23^ consistent with the reported inactivation of the wild-type *BHD* allele by somatic mutation or LOH in BHD associated renal tumours.^24^

Until recently the function of folliculin was unknown. Taking a genetic approach, Singh and colleagues demonstrated that *Drosophila* DBHD is required for male germline stem cell maintenance in the fly testis and functions downstream of the JAK/STAT and decapentaplegic signal transduction pathways.^25^ Recently, Baba and colleagues identified a novel folliculin-interacting protein, FNIP1, by co-immunoprecipitation studies in mammalian cells. They found that FNIP1 binds to 5′-AMP activated protein kinase, a negative regulator of mTOR suggesting that folliculin and its interacting partner may be involved in the AMPK and mTOR signalling pathways.^26^

Understanding the mutation spectrum as well as defining the clinical features of BHDS is important for diagnosis and *BHD* mutation screening, as well as for surveillance and treatment. In this study, we investigated new clinical features, *BHD* germline mutations, and potential genotype–phenotype associations in 51 families with BHDS.

## PATIENTS AND METHODS

### Patient recruitment and evaluation

All patients were evaluated at the National Institutes of Health (NIH) Clinical Center in consecutive order in a protocol approved by the Institutional Review Board of the National Cancer Institute (NCI). All members of families screened for BHDS who participated in this study gave written informed consent. We recruited BHDS families through referrals from the 11 000 members of the American Academy of Dermatology and were also referred families for treatment of familial kidney cancer. Forty-four families were ascertained because of cutaneous signs of BHDS and seven families were referred to the Urologic Oncology Branch for evaluation and treatment of familial kidney cancer. Medical histories were obtained (fibrofolliculomas, spontaneous pneumothorax and renal tumours) and physical examinations were performed. History of other neoplasms and pneumothorax in the patient or family were also recorded. Each individual had a detailed dermatologic examination and skin biopsies were obtained of selected lesions. Computed tomography (CT) scans of the chest and abdomen were used to screen for pulmonary and renal abnormalities respectively, as previously described.^6^ Outside medical records and pathology reports were reviewed, and tissue specimen blocks and slides were reviewed by NIH pathologists. Blood was drawn for *BHD* mutation analysis.

### *BHD* mutation analysis

DNA was extracted from peripheral blood leucocytes according to standard procedures. Methods for identification of exon/intron boundaries and high throughput DNA sequencing were as previously described.^4^ At least 160 unrelated control individuals were examined for each disease associated sequence variant. Insertion and deletion mutations were confirmed by subcloning using Topo Cloning Kit (Invitrogen) and sequencing.

### Statistical analysis

Data collected from BHDS patients included: presence of fibrofolliculomas, presence of renal tumours, gender, history and number of pneumothoraces, and presence of lung cysts. Analyses with respect to dichotomous parameters and their relationship to presence or absence of a pneumothorax, renal tumour or fibrofolliculomas were done using a Fisher’s exact test. Mehta’s modification to Fisher’s exact test was used to compare unordered categorical data to a dichotomous parameter.^27^ An exact Cochran–Armitage test for trend was used to determine the association between the number of pneumothoraces considered in ordered categories, and the presence or absence of a family history of pneumothoraces.^28^ All p values are two-sided and were not adjusted for multiple comparisons. However, in view of the exploratory nature of the study and the large number of comparisons performed, only p values <0.01 should be considered as being associated with statistically significant tests, while those between 0.01 and 0.05 would be considered trends.

### Multiple sequence alignment of the folliculin protein

We analysed evolutionary conservation by comparing human folliculin against the orthologous folliculin sequences of chimpanzee, monkey, mouse, rat, dog, horse, cow, chicken, frog, zebrafish and sea urchin from the National Center for Biotechnology information (NCBI) protein database.^29^ The ClustalW Multiple Sequence Alignments algorithm (European Bioinformatics Institute) was used to evaluate sequence conservation.^30^

### Literature review

We performed an electronic search from 2001 to 2007 designed to capture all reported cases of BHDS with *BHD* (also known as FLCN) germline mutations. Cases were excluded if mutations were somatic (not shown in a second tissue like normal kidney or peripheral blood). However, cases were still included in the review even if the method of mutation detection was not included. In total, 11 articles were ultimately included.^5^^–^15 Cases were cross-referenced in order to avoid duplication of cases. Only one case was reported twice.^7^ ^8^ Germline *BHD* mutation, presence of fibrofolliculomas, renal tumours, lung cysts, and history of pneumothorax, regardless of radiological methods, were recorded.

## RESULTS

### BHD mutation analysis

**Figure 1:**
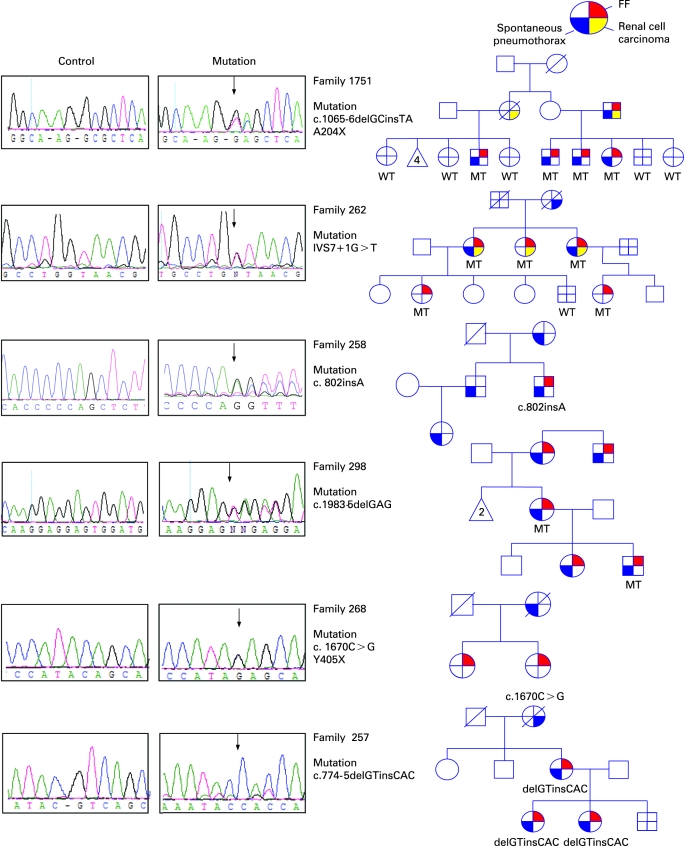
*BHD* mutations newly characterised in families with Birt–Hogg–Dubé syndrome (BHDS). Sequencing chromatograms of genomic DNA from control subjects and patients are shown on the left. The arrows indicate the position of the identified nucleotide changes. The corresponding pedigrees are shown on the right. FF, fibrofolliculoma; MT, mutant; WT, wildtype.

Ninety-eight individuals from 58 families were screened for *BHD* mutations. Using direct sequencing analysis we identified *BHD* germline mutations in 89 individuals from 51 families with BHDS (88% (51/58) *BHD* mutation detection rate). Direct sequence analysis of the 14 coding exons and splice site junctions of *BHD* revealed 23 unique germline mutations including: one missense, eight deletions, four insertions, one insertion/deletion, three nonsense, and six splice site mutations (table 1). Of these 23 germline mutations, 13 were novel and consisted of: three deletions (c.602delA, c.1707delC, c.1983-5delGAG), two insertions (c.802insA, c.1741insA), an insertion/deletion (c.774-5delGTinsCAC), four splice site mutations (IVS4-2 A>G, IVS7+1 G>T, c.1755 G>A, IVS12+1 G>A), one missense (c.1978A>G [K508R]) and two nonsense (c.1065-6delGCinsTA, c.1670C>G) (table 1, fig 1). Splice site and missense mutations were not detected in more than 160 normal individuals. All insertions and deletions were confirmed by subcloning. The c.1755 G>C and c.1755 G>A affect the last coding nucleotide of exon 11 resulting in aberrant mRNA splicing. Splicing alteration of exon 11 as a consequence of this mutation has been previously demonstrated.^7^

**Table 1:** Spectrum of germline *BHD* mutations and phenotypic features of 51 National Cancer Institute families with Birt–Hogg–Dubé syndrome

Family ID	Mutation analysis					Phenotype in family members					
	Intron	Exon	Nucleotide change	Codon location	Mutation type	No. clinical affected	No. with FF	Other skin lesions	No. with renal tumours	No. with lung cysts	No. with pneumo-thorax
251		4	c.514delT†	F20	Deletion	1	1	TD, BCC	0	0	0
252		4	c.602delA	E49	Deletion	1	1		0	1	1 (n = 3)
253	4		IVS4-2A>G		Splice site	1	1		0	1	1 (n = 1)
240	4		IVS4-2A>G		Splice site	10	9	TD, PFF	1	4	0
254	4		IVS4-2A>G		Splice site	1	1	TD	1	1	0
255	4		IVS4-2A>G		Splice site	1	1	PFF	0	1	1 (n = 2)
256*		5	c.751delA	D99	Deletion	1	0	PFF	1	1	1 (n = 5)
1798		5	c.774-5delGTinsCAC	V107	Deletion/ insertion	1	1	TD	0	1	0
257		5	c.774-5delGTinsCAC	V107	Deletion/ insertion	4	3	PFF, SH	0	4	0
258		5	c.802insA	Q116	Insertion	1	1	LM	1	1	1 (n = 17)
259		6	c.1039delG†	G195	Deletion	1	1		1	1	0
260		6	c.1039delG†	G195	Deletion	1	1	SH, PFF	0	1	1 (n = 1)
1751		6	c.1065-6delGCinsTA	A204X	Nonsense	4	4	AF, BCC	0	4	2 (n = 2)
261	7		IVS7+1 G>T		Splice site	1	1	L	1	1	1 (n = 3)
262	7		IVS7+1 G>T		Splice site	5	4	AF, DFSP, LS, TD	4	5	1 (n = 1)
263*	7		IVS7+1 G>T		Splice site	1	1		1	1	1 (n = 1)
264		9	c.1378-1405dup	T317	Insertion	1	1	PFF	1	1	0
265		9	c.1378-1405dup	T317	Insertion	2	2	PFF	1	2	2 (n = 4)
266		9	c.1378-1405dup	T317	Insertion	1	1	TD	0	1	0
267	9		IVS9+2 T>G		Splice site	1	1		0	1	0
268		11	c.1670C>G	Y405X	Nonsense	1	1	DF	1	1	0
269		11	c.1707delC	L418	Deletion	1	0	AF, SH, SCC	0	1	0
270		11	c.1733insC	H429	Insertion	1	1	MM, L	1	0	0
271		11	c.1733insC	H429	Insertion	2	2	TD, PFF, CTN	0	1	0
272		11	c.1733insC	H429	Insertion	1	1	BCC, PFF	0	1	0
273		11	c.1733insC	H429	Insertion	1	1	PFF	0	1	0
274		11	c.1733insC	H429	Insertion	1	1	PFF, SH	0	1	1 (n = 4)
275		11	c.1733insC	H429	Insertion	2	2		1	2	1 (n = 5)
276		11	c.1733insC	H429	Insertion	1	1	BCC	0	0	0
277		11	c.1733insC	H429	Insertion	2	2	PFF	0	2	0
278		11	c.1733insC	H429	Insertion	3	2	PFF	0	2	0
279*		11	c.1733insC	H429	Insertion	2	2		2	2	1 (n = 1)
280		11	c.1733insC	H429	Insertion	3	2	AF, CTN	0	3	3 (n = 5)
281		11	c.1733insC	H429	Insertion	2	2	AF	0	2	1 (n = 5)
282		11	c.1733insC	H429	Insertion	1	1		1	1	0
283		11	c.1733insC	H429	Insertion	3	2		1	3	1 (n = 1)
284		11	c.1733delC	H429	Deletion	4	3	L, MM	1	4	3 (n = 4)
285*		11	c.1733delC	H429	Deletion	1	1		1	1	1 (n = 7)
286		11	c.1733delC	H429	Deletion	1	1		0	1	0
287		11	c.1733delC	H429	Deletion	1	0	TD	1	1	1 (n = 1)
288		11	c.1733delC	H429	Deletion	2	2	AF	2	2	0
289		11	c.1741insA	H429	Insertion	1	1	BCC, SCC	0	1	1 (n = 3)
290*		11	c.1755G>C	E434Q	Splice site	1	1		1	0	0
291*		11	c.1755G>A	E434K	Splice site	2	1	PFF	1	2	1 (n = 4)
292		12	c.1834-5delTC	L460	Deletion	1	1		1	1	1 (n = 1)
293		12	c.1844C>G	Y463X	Nonsense	2	2	AF	0	2	1 (n = 2)
294		12	c.1844C>G	Y463X	Nonsense	1	0	PFF, AF	0	0	0
295		12	c.1844C>G	Y463X	Nonsense	1	1	AF	1	1	1 (n = 4)
296	12		IVS12+1G>A		Splice site	1	1	TD, PFF	0	1	1
297*		13	c.1978A>G	K508R	Missense	1	0	0	1	0	0
298		13	c.1983-5delGAG	E510	Deletion	2	1	AF	0	2	2 (n = 4)
Totals: 51 families						89	75		30	75	

AF, angiofibroma; BCC, basal cell carcinoma; CTN, connective tissue nevus; DFSP, dermatofibrosarcoma protuberans; FF, fibrofolliculoma; L, lipoma; LM, cutaneous leiomyoma; MM, malignant melanoma; PFF, perifollicular fibroma; LS, cutaneous leiomyosarcoma; SCC, squamous cell carcinoma; SH, sebaceous hyperplasia; TD, trichodiscoma.

FF diagnosis is based on histological diagnosis only.

*Families ascertained on the basis of kidney tumours. †In these cases of deletions in a mononuclear tract, the last nucleotide is given. The c.514delT mutation was formerly reported as c.513delT and the c.1039delG was formerly reported as c. 1036delG.

*BHD* mutations were identified affecting only translated exons 4, 5, 6, 9, 11, 12 and 13 (table 1). Exon 11 was the most frequent site of mutation representing 47% (24/51) of the BHDS families. A total of 19 families had a germline *BHD* mutation in the mononucleotide tract of eight cytosines (c.1733–1740), the mutation “hotspot” in exon 11. Seventy-four per cent (14/19) had a cytosine insertion (c.1733insC) and 26% (5/19) had a cytosine deletion (c.1733delC). Three of the four families with mutations in exon 12 shared a common mutation (c.1844C>G). Four families had a *BHD* mutation in exon 5. Exons 9 and 6 were each the site of mutations in three families. All three families with mutations in exon 9 had the 28-bp duplication (c.1378-1405dup). Two families had unique mutations in exon 4 and two families had a unique mutation in exon 13. Nine families had intronic mutations that were predicted to cause exon skipping: three in intron 7 (IVS7+1G>T), four in intron 4 (IVS4-2 A>G), one in intron 9 (IVS9+2 T>G) and one in intron 12 (IVS12+1 G>A) (table 1). The most 5′ mutation (c.514delT) occurred in exon 4 (20 aa from the initiation codon) and is predicted to truncate the protein 34 amino acids downstream. The most 3′ mutation (c.1983-5delGAG) occurred in exon 13 and it is predicted to produce a deletion of a single amino acid at position 510.

**Figure 2:**
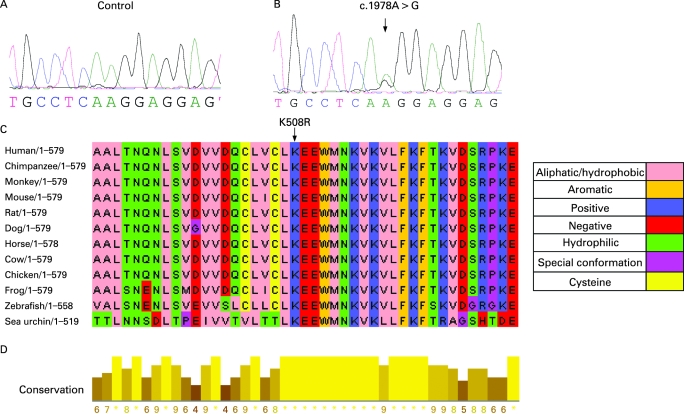
Novel *BHD* missense mutation (K508R). (A) Genomic sequence of control. (B) Genomic sequence of BHDS patient with the c.1978A>G (K508R) mutation. (C) Multiple sequence alignment. The amino acid residues are coloured according to their chemical properties. (D) Degree of conservation. The numerical index reflects the degree of conservation of the physical–chemical properties in the alignment. Star (*) indicates amino acids 100% identical in the alignment (highest score). The next most conserved group is composed of substitutions in amino acids with the same physical chemical class.

The novel missense mutation (K508R) was also confirmed by a CLIA approved laboratory and it was not present in DNA from 160 unrelated control individuals. The K508R mutation leads to a change from lysine to arginine at amino acid position 508 (fig 2). ClustalW multiple protein sequence alignments showed that amino acid K508 of folliculin is highly conserved in chimpanzee, horse, mouse, rat, dog, cow, chicken, zebrafish and sea urchin (fig 2). In addition, a block of continuous high conservation was shown between amino acids L507 to V515. The conservation across species suggests an important biological role for K508 amino acid in folliculin.

### Clinical features

Patients’ clinical characteristics are listed in table 1. Our cohort included a total of 89 individuals from 51 families with *BHD* mutations. There were 37 men and 52 women with a median age of 54 years. The number of affected individuals in a family ranged from 1 to 10. Thirty-three families had only one member affected with BHDS who participated in the study.

#### Cutaneous findings

Ninety per cent (46/51) of families with BHDS had individuals with multiple 1–5 mm white or skin coloured papules distributed over the face, neck and/or upper trunk and a histologically confirmed fibrofolliculoma (FF). Fifty-seven per cent (26/46) of BHDS families with a histologically confirmed FF also had individuals with a second histologically confirmed cutaneous lesion associated with BHDS including: angiofibroma (AF) or trichodiscoma (TD) (12 families), perifollicular fibroma (PFF) (11 families), and both TD and PFF (three families). In 10% (5/51) of BHDS families the clinical diagnosis of FF was not confirmed histologically. However, four of these families had at least one individual with a histologically proven TD or AF (one individual in each of two families), or PFF (one individual in one family), or both an AF and a PFF (one individual in one family). One family had no individuals with FF, PFF, TD or AF. All of these individuals also had a germline *BHD* mutation. In addition, three of these families had individuals with a history of spontaneous pneumothorax and/or lung cysts supporting the clinical diagnosis of BHDS.

Other dermatologic conditions histologically confirmed in individuals with a germline *BHD* mutation included: basal cell carcinoma (four families), connective tissue nevus (two families), sebaceous hyperplasia (two families), squamous cell carcinoma (SCC) (one family), and malignant melanoma (two families) (table 1). Two families had cutaneous soft tissue tumours: family 258 had one individual with a cutaneous leiomyoma (LM) and family 262 had one individual with a dermatofibrosarcoma protuberans (DFSP) and another with a cutaneous leiomyosarcoma (LS).

#### Kidney tumour findings

Overall 34% (30/89) of individuals and 49% (25/51) of families with BHDS had kidney tumours (table 1). Men (13 cases) developed renal tumours about as often as women (17 cases). Patients had either bilateral, multifocal or unilateral renal tumours (>0.5 cm lesion).

Among 25 families with kidney tumours, 18 (72%) were recruited based on cutaneous manifestations suggestive of BHDS and/or the histologic diagnosis of fibrofolliculoma and seven (28%) were recruited based on kidney tumours. Approximately 41% (18/44) of families presenting with cutaneous manifestations had kidney tumours. We observed that 88% of BHDS families with renal tumours had only one individual with renal tumours even after screening multiple *BHD* gene mutation carriers for renal tumours with abdominal CT or magnetic resonance imaging (MRI) of the kidneys.  Only three families had multiple living individuals affected with kidney tumours. Family 279 and family 288 each had two living cases and family 262 had four cases. An individual from family 297 with a germline *BHD* mutation presented with bilateral multifocal renal oncocytomas as the only sign of BHDS. He had no skin papules, history of pneumothorax or lung cysts on CT imaging.

#### Pulmonary findings

Eighty-eight per cent (45/51) of the BHDS families and 84% (75/89) of BHDS patients had lung cysts on CT imaging (table 1). Fifty-three per cent (27/51) of BHDS families and 38% (34/89) of individuals with BHDS had a history of spontaneous pneumothorax. Most patients with a history of pneumothorax had lung cysts by chest CT imaging (table 1). Thirty-four individuals had a total of 92 spontaneous pneumothoraces. Nineteen patients had two pneumothoraces, three patients had four pneumothoraces, three had five pneumothoraces, and one experienced seven pneumothoraces (table 1).

#### Family history

We found that 18 of 31 patients (58%) with a family history of pneumothorax developed pneumothoraces compared to 16 of 57 patients (28%) without this family history (p = 0.011). Among patients with BHDS, there was a statistically significant trend toward having a greater number of pneumothoraces if the patients had a family history of pneumothoraces compared to the number of pneumothoraces in patients who did not have this family history (p = 0.0022, by an exact Cochran–Armitage trend test). However, family history of pneumothorax was not associated with an increased risk of kidney tumours. Twelve of 34 patients with a family history of pneumothoraces had kidney tumours compared to 18 of 55 without this family history (p = 0.82).

BHDS patients with a family history of kidney tumours had a statistically significantly increased probability of having renal tumours, with 16 of 28 (57%) individuals having these tumours among those with a family history for this condition as compared to 14 of 61 (23%) without a family history of kidney tumours (p = 0.0032). However, we observed that a family history of renal tumours was not associated with an increased risk of lung cyst (p = 0.32) or pneumothrorax (p = 0.16).

#### Variable expression of phenotypic manifestations

Variable phenotypic expression may be observed among families and within members of BHDS families (table 1). Among the 51 families with BHDS: 25% (13/51) had FFs, a history of spontaneous pneumothorax and renal tumours; 24% (12/51) had both FFs and a history of spontaneous pneumothorax but no renal tumours; 18% (9/51) had both FFs and kidney tumours but no individuals with a history of pneumothorax; 24% (12/51) had FFs but no renal tumour or history of pneumothorax; 4% (2/51) had no FFs, renal tumour or a history of pneumothorax; 2% (1/51) had renal tumour but no FFs or a history of pneumothorax; and 4% (2/51) had renal tumours and a history of pneumothorax but no FFs (table 1).

#### Other clinical findings

Among the BHDS patients in the present study, two had parotid gland oncocytomas diagnosed at ages 20 and 39 years. Non-renal cancers that occurred among the BHDS patients included two cases of colon cancer and thyroid cancer, respectively; and single cases of squamous cell carcinoma (SCC) of the head and neck, Hodgkin’s disease, uterine cancer, prostate cancer, breast cancer, and SCC of the cervix. Other tumours were rhabdomyoma and an adrenal mass (table 1).

### Genotype–phenotype correlations

#### BHD mutation hotspot: c.1733insC and c.1733delC

There was no association between *BHD* mutation hot spot versus all other *BHD* mutations, and presence of FFs (p = 0.78), presence of lung cysts (p = 0.76) or presence of pneumothoraces (p = 1.0). We also investigated for genotype–phenotype correlation among c.1733insC and c.1733delC *BHD* mutation carriers. The frequency of individuals with histologically proven FFs was similar among c.1733insC *BHD* mutation carriers (88% (22/25)) and c.1733delC *BHD* mutation carriers (78% (7/9)) (p = 0.59). Lung cysts were detected similarly among c.1733insC (84% (21/25)) and c.1733delC *BHD* mutation carriers (100% (9/9)) (p = 0.55). History of spontaneous pneumothorax was somewhat more common among c.1733delC (56% (5/9)) mutation carriers than among c.1733insC carriers (32% (8/25)) but not statistically significant (p = 0.25). Renal tumours were more common among c.1733delC (56% (5/9)) than c.1733insC (24% (6/25)) *BHD* mutation carriers but not statistically significant (p = 0.11). To explore further potential genotype–phenotype correlations we combined the *BHD* mutation hotspot data from our present and previous study.^6^ Our analyses showed that c.1733insC mutation carriers and c.1733delC mutation carriers had similar frequencies of FFs (85% (66/78) vs 84% (27/32) p = 1.00), lung cysts detected on thoracic CT scans (86% (56/65) vs 84% (22/26) p = 1.00), spontaneous pneumothorax (36% (31/87) vs 40% (14/35) p = 0.68) and renal tumours (29% (19/65) vs 24% (6/25) p = 0.79). Therefore, in general there were no statistically significant phenotypic differences among c.1733insC and c.1733delC carriers in BHDS families seen at NCI.

#### Other types of mutations

There was no association between *BHD* mutation status (no mutation vs mutation), mutation types ((insertion, deletion, nonsense, missense and splice site) (insertion vs other types of mutation), (deletion vs other types of mutation), (nonsense vs other types of mutation), (splice site vs other types of mutation), (missense vs other types of mutation), (frameshift vs other types of mutation), (intron vs exon mutations)), and cutaneous affected ((FF vs no FF) or (FF vs TD and/or PFF)), lung cyst (lung cyst vs no lung cysts ) or pneumothorax (history of pneumothorax vs no history of pneumothorax) in our cohort of patients.

### Review of published reports of *BHD* germline mutations

We reviewed cases with published *BHD* germline mutations and general clinical characteristics associated with BHDS.^5^^–^15 We previously reported 177 patients (88 men and 89 women) with *BHD* mutations from 51 BHDS families.^6^ Excluding the BHDS patients reported by NCI,^6^ a total of 85 individuals with *BHD* germline mutations have been reported including 35 women, 41 men and nine individuals in whom the gender was unspecified ranging from 12–77 years of age^5^ ^7^^–^15 (table 2).  The number of affected individuals in a family ranged from 1–12.

**Table 2:** Summary of reviewed reports with *BHD* germline mutations and general clinical characteristics, excluding National Cancer Institute reports

Reference	No. of patients	Clinical FF (Bx FF) (pos/neg/unk)	Renal tumour med Hx, and/or CT or MRI (yes/no/unk)	Lung cysts (pos/neg/unk)	Hx pneumo (yes/no/unk)	Germline mutations
Khoo *et al* 2002^5^	21	Pos 16/21 (16 Bx+)	Yes 2/15; Unk 6	Unk 21/21	Yes 8/21	c.1733delC, c.1733insC
Van Steensel *et al* 2007^7^	6	Pos 6/6 (6 Bx+)	No 5/5; 1 Unk	Neg 5/5	Yes 1/5	c.1863-1873delGGGAGCCCTGT
				Ukn 1	1 Unk	c.1755G>C, c. 875delC,
						c.1733insC, IVS10-2 A>G
Leter *et al* 2008^8^	23	Pos 18/23 (18 Bx+)	Yes 2/25	Ukn 25/25	Yes 4/25	c.1065-6delGCinsTA,
		2 Ukn				c.1733insC, c.875delC, c.1110dupG
						c.1756-7del11;1778delCinsGA
						c.IVS 6-1 G>A
Gunji *et al* 2007^9^	5	Neg 5/5	No 5/5	Pos 5/5	Yes 5/5	c. IVS5-1delgtccctccag,
				HRCT		c.1795 insCCACCCT, c.1733insC
						c.1988delGATG, c.857delC
Graham *et al* 2005^10^	11	Ukn 11/11	No 11/11	Pos 6/6	Yes 10/10	c.1398G>T, c.1884C>T
				Ukn 5	1 Ukn	
Painter *et al* 2005^11^	12	Unk 12/12	No 12/12	Pos 12/12	Yes 5/12	c.690-3delTCGG
Kawasaki *et al* 2005^12^	2	Pos 2/2 (2 Bx+)	No 2/2	Unk 2/2	No 2/2	c.1733insC
Lamberti *et al* 2005^13^	1	Pos 1/1 (Bx+)	Ukn	Unk	Ukn	c.1733insC
Bessis *et al* 2006^14^	2	Pos 1/2 (1 Bx+)	No 2/2	1 Neg; 1 Unk	Yes 1/2	c.458delG
Murakami *et al* 2007^15^	1	Ukn 1	Yes	Neg	No	c.1733insC
				Chest CT		
**Total**	85*	43/59 Pos(43 Bx+)	Yes 5/77	23/30 Pos	Yes 33/82	
		Ukn 26	Ukn 8	Ukn 55	Ukn 3	

Bx, biopsy; CT, computed tomography; FF, fibrofolliculoma; HRCT, high resolution computed tomography; Hx, medical history; MRI, magnetic resonance imaging; Neg, negative; Pos, positive; Ukn, unknown.

*One case was reported twice.^7^ ^8^

#### BHD germline mutations

Excluding our previously reported 22 *BHD* germline mutations,^4^ ^6^ 18 unique *BHD* germline mutations have been reported by other investigators including: seven deletion, three insertion, three nonsense, and five splice-site mutations (table 2).^5^ ^7^^–^15 Of a total of 40 *BHD* germline mutations reported to date, only four mutations (c.1065-6delGCinsTA, c. 1733insC, c. 1733delC and 1884C>T) were reported by both the NCI BHDS group and other investigators.^4^^–^9 12^–^14 A family with the c.875delC mutation was described in two different studies.^7^ ^8^ *BHD* mutations have been reported affecting all translated exons,^4^^–^14 with the exception of exons 8 and 10.^4^^–^15 Exon 11 was the most frequent site of mutations (c.1733insC/1733delC, c.1755G>C) present in 125 individuals from 40 unrelated BHDS families.^4^^–^9 12^–^14 The hot spot mutation (c.1733insC or c.1733delC) was the most common *BHD* mutation reported to date in a total of 124 individuals from 39 BHDS families.^4^^–^9 12^–^14 Seventy-nine individuals from 28 families (18 NCI and 10 other groups) were reported to have the c.1733insC mutation and 45 individuals from 11 families (nine NCI and two other groups) were reported as having the c.1733delC mutation.^4^^–^9 12^–^14

The most 5′ BHD mutation reported occurred at nucleotide 454 (c. 458delG) in exon 4 affecting the initiator codon of *BHD*.^14^ Other *BHD* germline mutations have been previously reported in exon 4: c.690delTCGG predicted to result in a termination codon 50 amino acids downstream^11^ and c.514delT predicted to result in a premature termination codon at codon 34.^6^ The most 3′ mutation reported occurred at nucleotide 2034 (c.2034 C>T) in exon 14 and was predicted to produce a premature termination codon at codon 527.^6^ No additional *BHD* mutations have been reported in exon 14.

#### Clinical features

In the previously reported NCI BHDS cohort, 143 patients with *BHD* mutations had their skin papules biopsied and evaluated histologically. Of these 143 cases, 122 (85%) were diagnosed with a histologically proven FF.^6^ Excluding the NCI BHDS patients reported,^6^ 73% (43/59) of individuals with germline mutations in *BHD* had a histologically confirmed FF (table 2).^5^ ^7^^–^15 In five individuals with germline *BHD* mutations, FF was not confirmed histologically but they had a histologically proven trichodiscoma.^8^

We previously reported 35 patients with *BHD* germline mutations who developed renal tumours.^6^ The frequency of renal tumour among patients with *BHD* germline mutations seen at the NIH clinical centre or in field trips was 22% (35/162) or 20% (35/177) of all patients of whom blood was collected and screened for mutations.^6^ Two BHDS patients died of metastatic kidney cancer after radical nephrectomy.^31^ One patient underwent bilateral nephrectomies for multiple tumours and received a kidney transplant. Pathological examination revealed that his largest tumour was an 8 cm clear cell renal cell carcinoma (RCC) and the other tumours had multiple histologies. This patient had biopsy proven paraspinal metastasis (clear cell histology) 56 months after his initial surgery and he died shortly thereafter. The other patient had an 8 cm predominantly clear cell RCC with areas of tubular papillary and chromophobe histology that invaded the perinephric fat. This patient had retroperitoneal recurrence 5 months after surgery, eventually developed osseous metastases and died 20 months postoperatively.^31^

Excluding previous NCI BHDS reports,^6^ overall in the literature five of 77 (6.5%) individuals with *BHD* germline mutations had a recorded medical history of kidney tumours and/or renal tumour on renal imaging (table 2). The age at diagnosis of a renal tumour ranged from 20–55 years.^5^ ^8^ ^15^ Khoo and co-workers reported a 65-year-old BHDS patient with a history of right nephrectomy at age 20 for clear cell RCC, and an additional patient who at age 35 had a nephrectomy for an 8 cm “malignant oncocytoma” infiltrating the capsule.^5^ The latter patient developed metastatic disease involving the right upper lobe of the lung which upon thoracotomy revealed metastatic RCC with papillary structures. Microscopic re-examination of tumour tissue revealed unclassified RCC, Fuhrman grade 4.^5^ Leter and co-workers reported an individual with a history of RCC who had unilateral mixed papillary and clear cell RCC at age 39 and a year later died due to metastatic disease.^8^ In addition, Murakami *et al* reported a 55-year-old who at the time of nephrectomy had a 9.5 cm single solid mass, an inferior vena cava tumour thrombus and lung metastasis.^15^ Microscopic examination of renal tumour showed tubular and tubulo-papillary areas with mainly eosinophilic neoplastic cells and focally clear cells. This patient died of metastatic disease 27 months after nephrectomy. Of 50 individuals with a germline *BHD* mutation who were screened for kidney tumours by other investigators,^5^ ^7^^–^15 only one had a renal tumour detected on baseline imaging (table 2).^8^ This patient was a 40-year-old woman with histologically confirmed trichodiscoma who was referred by the dermatologist for renal imaging. Renal work-up revealed RCC with elements of oncocytoma.

In our previous study, we found that 33% (57/171) of patients with *BHD* germline mutations for whom medical history was available reported a history of spontaneous pneumothorax and 87% (95/109) of BHDS who were screened by chest CT scan had one or more lung cysts.^6^ Excluding the BHDS patients reported by NCI,^6^ 77% (23/30) of patients reported with *BHD* germline mutations had lung cysts on CT imaging and 40% (33/82) of patients reported a history of spontaneous pneumothorax (table 2).^5^ ^7^^–^15

## DISCUSSION

**Figure 3:**
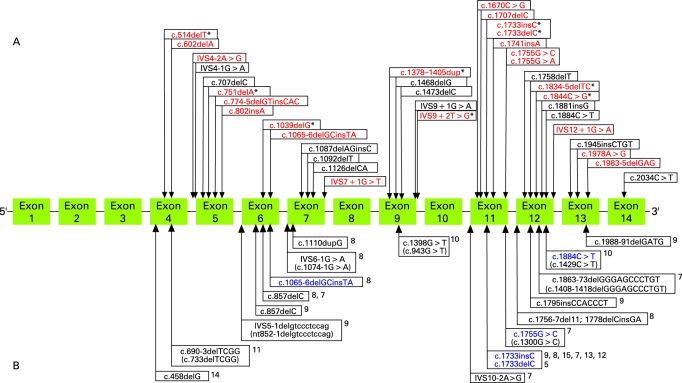
*BHD* germline mutation reported by the National Cancer Institute (NCI) group and other investigators. (A) Illustration of the 55 germline *BHD* mutations identified by the NCI. The 23 unique *BHD* mutations identified in the present study are shown in red. The star indicates mutations identified in both the present study and the previous NCI study.^6^ The 22 unique mutations previously reported by NCI are shown in black.^6^ (B) Lower panel shows the 18 germline *BHD* mutations reported by other investigators.^5^ ^7^^–^15 Mutations shown in blue were identified by both the NCI group and other investigators. The parenthesis show the original nomenclature used in the original reports.

We report the clinical findings in 50 new families with BHDS and 13 novel *BHD* germline mutations. We identified *BHD* mutations in 88% of BHDS families which is slightly higher than our previously reported mutation detection rate of 84%.^6^ In combination with our previous reports,^4^ ^6^ to date we have reported 102 families and 36 unique *BHD* germline mutations including 21 insertion/deletions, eight putative splice site, six nonsense and one missense mutation (fig 3). Including the present study and all cases worldwide, 53 unique germline *BHD* mutations have been reported affecting all translated exons (4-14), with the exception of exons 8 and 10 (4-15). In this investigation, we report the first germline missense mutation (K508R) in *BHD* (fig 2). Using sequence alignment, we found that the lysine508 amino acid in folliculin is conserved in vertebrate and invertebrate species suggesting functional significance. The K508R mutation was present in a patient with bilateral multifocal renal oncocytomas, a phenotype commonly seen in BHDS, suggesting that it is a disease-causing mutation. This mutation was not present in DNA from 160 unrelated control individuals. Therefore, although uncommon, germline *BHD* missense mutations may occur in BHDS. This is not surprising since a missense mutation in exon 7 (H255R) in the canine *BHD* ortholog is the disease-associated germline mutation in hereditary multifocal renal cystadenocarcinoma and nodular dermatofibrosis in German shepherd dogs.^21^ The H255R mutation confers an amino acid change in a highly conserved histidine of folliculin.

Including the present study, 70 patients with *BHD* germline mutation and kidney tumours have been reported in the literature.^5^ ^6^ ^8^ ^15^ In the present NCI study, the age at diagnosis of kidney tumours was similar to our previous reports^6^ ^31^ and reports by other investigators.^5^ ^7^^–^15 Even though in the present study we did not identify BHDS patients with metastatic kidney cancer, only five BHDS patients with *BHD* mutations who died of metastatic kidney cancer have been reported to date.^5^ ^8^ ^15^ ^31^ These individuals with metastatic kidney cancer had tumours with clear cell, tubulo-papillary and/or papillary histologic features. These histologic features are slightly different from most kidney tumours associated with BHDS. BHDS is associated with a unique histological spectrum of bilateral and multifocal kidney tumours ranging from hybrid oncocytic (67%) to chromophobe RCC (23%) to oncocytic (3%).^31^ Clear cell RCC (3%) has also been reported in a few BHDS cases.^31^ Prospective studies with a larger number of patients are needed to determine whether the different renal tumour histopathologic subtypes associated with BHDS have different malignant potentials. It is of interest that even though 95% of patients with *BHD* germline mutations and kidney tumours have been reported by the NCI, to our knowledge only two cases from the NCI group have developed metastatic kidney disease.^31^ In contrast, only a few BHDS patients with a *BHD* germline mutation have been reported by other investigators to have kidney tumours, and most (three of five) of these cases had metastatic disease.^5^ ^7^^–^15 These differences may be a reflection of the different ascertainment and screening methodologies for kidney tumour used by different research groups.

The present investigation confirms the high prevalence of kidney tumours in our BHDS families. Previously, we observed a sevenfold increased risk of renal tumours in patients with BHDS.^32^ In the present study, using a combined ascertainment in dermatologic and urologic oncology clinics at NCI, the overall prevalence of kidney tumours among individuals with germline *BHD* mutations was 34%. This frequency is higher than our previous report (23%)^6^ and much higher than the frequency of kidney tumours in BHDS cases combined from other investigators (6.5%). We also observed that BHDS patients with a family history of kidney tumours had a statistically significant risk of developing kidney tumours compared with BHDS patients without a family history of kidney tumours (p = 0.0015). The risk remained significant when we excluded cases ascertained on the basis of kidney tumour. Kidney tumour is also a major phenotypic feature with high penetrance in the naturally occurring BHDS animal models.^17^ ^18^ However, other genetic and environmental factors may also be involved in the development of kidney tumours in patients of BHDS and animal models.

Renal tumours associated with BHDS may have a higher impact on morbidity than on mortality. The presence of oncocytosis in the renal parenchyma at surgery or at postmortem examination of patients with BHDS is histologic evidence of the potential of kidney tumour development in BHDS patients. The diagnosis in a family member and surveillance of families with BHDS should lead to identification of patients at high risk of BHDS kidney tumours and to early detection and treatment. The use of nephron sparing surgery rather than radical nephrectomy decreases the morbidity of BHDS patients with renal tumours by preserving renal functional tissue. The diagnosis of BHDS is of critical importance in a patient presenting with kidney tumours since the management of these tumours is different from sporadic kidney tumours.^31^ Somatic *BHD* mutations or LOH at the *BHD* locus have been detected in 70% of RCCs from BHDS patients.^24^ The high frequency of second hits in *BHD* and *bhd* supports that it is a tumour suppressor gene. Inactivation of both *BHD* alleles occurs in several histologic subtypes of RCC, suggesting that inactivation of *BHD* occurs at an early stage of renal tumorigenesis.^24^

In the present study, we found that 84% of patients with *BHD* germline mutations have pulmonary cysts on chest CT. This is very similar to our previous study (89%)^6^ but slightly higher than other reports combined (77%)^5^ ^7^^–^15 In the present study we found that 38% (34/89) of individuals with a *BHD* germline mutation had a history of spontaneous pneumothorax. This is very similar to all other studies combined and our previous study (33%).^6^ In the present study, we also observed that patients with BHDS and a family history of pneumothorax had a statistically significant increased risk of pneumothorax when compared to BHDS patients without a family history of spontaneous pneumothorax. This supports previous reports of families with *BHD* mutations and a prominent and almost exclusive spontaneous pneumothorax inherited in an autosomal dominant fashion.^10^ ^11^ Most cases with lung phenotype without other cutaneous or renal manifestations probably are undetected cases of BHDS since they are from cross sectional studies and cases may develop other manifestations with time. Therefore, prospective follow-up clinical studies of this group of patients are needed to understand better how they fit within the spectrum of clinical manifestations of BHDS.

Previously, we reported a 50-fold increased risk for the development of spontaneous pneumothorax in patients with BHDS compared with family members unaffected by BHDS.^32^ The role of lung cysts in the mechanism leading to a spontaneous pneumothorax in BHDS had not been established. Lung cysts appear to be a precursor lesion leading to a spontaneous pneumothorax. Recently, we reported that total lung cyst volume, largest cyst diameter and volume, as well as every parameter associated with lung cysts which we evaluated, were significantly associated with pneumothorax in patients with BHDS.^33^ Haploinsufficiency may be responsible for the development of lung cysts, skin lesion and other hamartomas associated with BHDS. We have not observed increased mortality or progressive lung deterioration in BHDS patients with lung cysts or pneumothorax. The lung phenotype (history of spontaneous pneumothorax and lung cysts) associated with BHDS is a prominent feature that helps to distinguish BHDS from other inherited kidney cancer syndromes (von Hippel–Lindau syndrome, hereditary leiomyomatosis and renal cell cancer, and hereditary papillary renal cell carcinoma).^34^^–^37

We also report in this study for the first time families with germline mutations in *BHD* and angiofibroma, perifollicular fibroma or both angiofibroma and perifollicular fibromas as their only BHDS cutaneous phenotype. Our findings support that BHDS is associated with a spectrum of cutaneous hamartomas ranging from AF to PFF to FF. BHDS associated hamartomas should be distinguish it from other genodermatoses with an increased risk for internal malignancy.^38^ Recently, a *BHD* mutation was reported in one of three families with only trichodiscomas.^8^ However, two of the three families had an atypical presentation and most likely do not have BHDS. In addition, we previously reported additional individuals with *BHD* germline mutations and trichodiscomas.^6^ It is of interest that we identified two individuals with germline mutations in *BHD* who also had a history of malignant melanoma. Only one previous confirmed case of melanoma in a BHDS patient has been reported.^5^ We also identified a BHDS family in whom one sibling developed a DFSP and another sibling had a cutaneous leiomyosarcoma. These tumours are rare and not previously reported in BHDS. We also identified two additional individuals with parotid gland tumours for a total of six cases in our group of 102 BHDS families^6^ and a total of eight cases reported to date.^39^ Most of the other internal neoplasms identified in this study have been previously reported in patients with BHDS.^5^ ^40^^–^45 However, determining whether they are part of the clinical spectrum of BHDS remains to be investigated in further studies.

In this study, we investigated potential genotype–phenotype relationships in our patients with BHDS. In general, we found no associations between specific *BHD* mutations or mutation types (intron vs exon; frameshift, nonsense, missense) and clinical phenotype (FF, TD/AF, PFF, renal tumours, spontaneous pneumothorax, or lung cysts). Previously, we reported that individuals with an *BHD* IVS9+2 mutation had a higher frequency of kidney tumours compared to all mutation carriers.^6^ In this study, only one family had this same mutation, thus this observation could not be confirmed. It is possible that aberrant mutant BHD proteins produced by splice site mutations have functional consequences and lead to renal tumorigenesis.

Each of our independent studies and combined analyses showed that c.1733insC and c.1733delC mutation carriers had similar frequencies of FFs, lung cysts and pneumothoraces. In a previous study, we found that the frequency of renal tumours in c.1733delC carriers was lower than the frequency of renal tumours in c.1733insC carriers.^6^ However, analysis of combined data from both studies showed no significant differences in frequencies of renal tumours among both groups. Future studies with combined data from large cohorts of BHDS families should have enough statistical power to confirm our studies and evaluate other potential genotype–phenotype correlations.

The majority of *BHD* germline mutations identified in this investigation, both of our studies combined and all studies combined, are predicted to produce a C-terminally truncated folliculin (FLCN) resulting in loss of function.^4^^–^15 In this study, we report a mutation in a family in which a *BHD* mutation was not previously detected.^6^ Re-sequencing DNA from family 240, who linked to chromosome 17p11.2, revealed that affected haplotype carriers had a putative splice site mutation in the BHD gene within intron 4 (IVS4-2 A>G) (table 1). However, we did not identify sequence variations in the coding region and 3′ and 5′ regulatory regions of *BHD* in seven additional families despite two attempts using direct DNA sequencing. Future studies will include screening families for large deletions in *BHD*.

Recently, Baba and co-workers^26^ reported that FLCN interacts with FNIP1 through its C terminus. Therefore, germline mutations predicted to produce a C-terminally truncated FLCN would be unable to bind FNIP1. These investigators also showed that FLCN phosphorylation facilitated by FNIP1 is reduced by inhibitors of mTOR and AMPK activity implicating FLCN/FNIP1 in the AMPK and mTOR signalling pathways.^26^ Recently, Henske and co-workers deleted the BHD homolog in *Schizosaccharomyces pombe*.^46^ They showed that expression profiling revealed that six permease and transporter genes, known to be down-regulated in Deltatsc1 and Deltatsc2, were up-regulated in Deltabhd. They also showed that loss of Bhd sensitised yeast to rapamycin induced increases in permease expression levels, and rapamycin induced lethality in Deltabhd yeast expressing the hypomorphic Rhb1 allele.^46^ These results suggest that Bhd activates Tor2.

Advances in the diagnosis of BHDS using molecular genetic techniques has allowed us to expand the BHDS phenotypic spectrum of associated hamartomas and tumours, and demonstrate clinical heterogeneity both within and among BHDS families. Although we report a large group of families, our dataset is too small to examine genotype–phenotype correlations rigorously. However, this should be possible in the future by combining our clinical and *BDH* mutation data on clinically well described BHDS patients and families with that from other centres. Future characterisation of *BHD* mutations and genotype–phenotype correlations in BHDS may provide valuable insights into the molecular pathogenesis of BHDS. Future clinical studies and laboratory investigations using in vitro systems and animal models may help us to elucidate the sequence of pathogenetic events that lead to the clinical findings and the organ preference of involvement that we observe in BHDS.
